# A review of 3D printed patient specific immobilisation devices in radiotherapy

**DOI:** 10.1016/j.phro.2020.03.003

**Published:** 2020-03-20

**Authors:** Amirhossein Asfia, James I. Novak, Mazher Iqbal Mohammed, Bernard Rolfe, Tomas Kron

**Affiliations:** a School of Engineering, Faculty of Science, Engineering and Built Environment, Deakin University, Geelong, Victoria, Australia; b ARC Industrial Transformation Training Centre in Additive Bio-manufacturing, Brisbane, Queensland, Australia; c Loughborough Design School, Loughborough University, Loughborough, United Kingdom; d Peter MacCallum Cancer Centre, Melbourne, Victoria, Australia; e School of Applied Sciences, RMIT University, Melbourne, Victoria, Australia

**Keywords:** 3D printing, Additive manufacturing, Customisation, Head and neck cancer, Health technology, Systematic review

## Abstract

**Background and purpose:**

Radiotherapy is one of the most effective cancer treatment techniques, however, delivering the optimal radiation dosage is challenging due to movements of the patient during treatment. Immobilisation devices are typically used to minimise motion. This paper reviews published research investigating the use of 3D printing (additive manufacturing) to produce patient-specific immobilisation devices, and compares these to traditional devices.

**Materials and methods:**

A systematic review was conducted across thirty-eight databases, with results limited to those published between January 2000 and January 2019. A total of eighteen papers suitably detailed the use of 3D printing to manufacture and test immobilisers, and were included in this review. This included ten journal papers, five posters, two conference papers and one thesis.

**Results:**

61% of relevant studies featured human subjects, 22% focussed on animal subjects, 11% used phantoms, and one study utilised experimental test methods. Advantages of 3D printed immobilisers reported in literature included improved patient experience and comfort over traditional methods, as well as high levels of accuracy between immobiliser and patient, repeatable setup, and similar beam attenuation properties to thermoformed immobilisers. Disadvantages included the slow 3D printing process and the potential for inaccuracies in the digitisation of patient geometry.

**Conclusion:**

It was found that a lack of technical knowledge, combined with disparate studies with small patient samples, required further research in order to validate claims supporting the benefits of 3D printing to improve patient comfort or treatment accuracy.

## Highlights

• Investigating 3D printed immobilization devices.
• Comparing the 3D printed immobilization devices with traditional ones.
• Investigating the technical fabrication details of the articles.
• Introducing the scope, testing and results of the studied literatures.

## 1 Introduction

Radiotherapy is one of the most common treatments to stop the proliferation of cancerous cells, with almost half of cancer patients receiving radiotherapy during some period of their treatment [1], [2], [3]. Despite good therapeutic results, radiotherapy can have negative side effects, with the mechanism used to kill cancerous cells also capable of damaging healthy tissue. As a result, immobilisation devices are frequently used to minimise patient movements during radiation treatments, thereby ensuring the radiation dose is localised predominantly on the tumour site. This approach also limits exposure of healthy cells to radiation, while also allowing reproducibility of setup on a day-to-day basis [4]. Immobilisation devices can broadly be classified into two categories: invasive and non-invasive. Invasive fixation requires surgical fitting to the patient, and has been found to accurately hold a patient in position, for example cranial devices have been reported to hold patients within 1 mm of the desired position [5], [6]. However, invasive fixation suffers from issues of patient discomfort, potential for infection and the need for treatment to be conducted in one session.

Non-invasive fixation has become the preferred method of treatment, mitigating the issues of invasive fixation [7]. Considering the head and neck region, this method typically involves creating a custom-fitting mask made from a thermoplastic sheet moulded directly over the patient prior to treatment planning, with a thickness in the range of 1.6–3.1 mm [8]. The custom-fitting device can immobilise a patient through a close fitting with their anatomy and a secure fixture to a treatment table or other device. However, there remain several disadvantages to these masks including the accuracy of fitting being dependent on operator experience when thermoforming the mask, allowing at least some patients to maintain some motion during treatment [9]. Changes to facial geometry during the course of treatments due to weight loss can also be an issue with the fit of masks [8], and the mask fabrication process itself can be time-consuming, uncomfortable, claustrophobic and distressing for patients [10], [11], [12]. While some studies report a 1 mm accuracy using thermoplastic masks [5], [13], [14], [15], others argue that high accuracy comparable to invasive fixation is not possible [16].

In order to address these challenges, technologies such as 3D printing have seen increased research focus due to its ability to manufacture complex, customisable forms. Also known as Additive Manufacturing (AM) [17], [18], 3D printing is a layer-by-layer manufacturing process that directly produces an object from 3D digital data. AM has found clinical applications as varied as patient specific prosthetics manufacturing, anatomical models, implants, pharmaceutical research and organ and tissue creation [19]. In particular, AM has the capacity to reproduce the complexities of the human form [20], [21], [22], resulting in a new generation of patient tailored medical solutions. More recently, AM has been applied to upper body splint based applications [23], [24], [25], and there is growing interest for uses in radiotherapy treatments [10], [11]. As a result, it has gained the attention of researchers as a new method for producing non-invasive immobilisation devices utilising 3D patient models, such as those captured during Computed Tomography (CT) or Magnetic Resonance Imaging (MRI) [22], [26], [27], [28], combined with advanced Computer-Aided Design (CAD), 3D scanning [23], [25] and virtual planning software.

With the rapid developments of 3D printing technology, and the growing interest in its application to improve immobilisation devices, this review paper systematically analysed the academic literature on 3D printed immobilisers for radiation therapy published between January 2000 to January 2019. The primary goal was to provide researchers with an understanding of major developments, trends and opportunities in this field, providing insight into the advantages and disadvantages of the technology for the specific application of immobilisation. The secondary goal was to highlight gaps in the research for future enquiry.

## 2 Method

Initial scoping of literature began in February 2018. Thirty-eight databases and journals were included in the search in order to capture literature on the topic. These are listed alongside results in Supplementary Table S1. In February 2019 the final systematic literature review was conducted using the keywords: additively manufactured immobilisers, 3D printed thermoplastic masks, 3D printed masks, thermoplastic masks, head and neck thermoplastic masks, 3D printed immobilisers. A limitation was placed on the publication date of literature of January 2000 to January 2019 due to initial scoping search results and the growth of AM technology, which was predominantly used only for prototyping prior to 2000.

Manual screening of the titles and abstracts was performed to include only papers consistent with the application of 3D printing techniques for the creation of immobilisation devices used in radiotherapy treatments. Given the breadth of the search this meant many results were excluded due to various factors, including the use of 3D printing for purposes other than immobilisation, the use of 3D printing to prototype a concept before it was manufactured using another technology, or medical devices such as masks that were not additively manufactured. Duplicates were also removed.

Final screening was performed through reading of full-text papers and removing any that did not specifically discuss 3D printed immobilisation devices for radiotherapy treatments. Results were analysed by all authors to confirm suitable literature selection. Key information from each article was recorded in a spreadsheet to allow comparison, with categories including the type and number of test subjects, the specific part of the body examined and type of immobilisation, the objectives of the study, and the testing techniques. Technical fabrication details were also recorded including the type of printer, the utilised materials and the implemented software during the design procedure.

## 3 Results

A total of 4152 papers were identified through the search. Supplementary Table S1 identifies the 38 databases and journals, along with the number of initial results collected. Following the exclusion strategy visualised in Supplementary Fig. S1, 4080 results were removed, and a further 54 duplicates were also removed. The result was 18 papers that were appropriate for this study and analysed in detail.

As shown in Supplementary Fig. S2, only one journal article was dated before 2014, occurring in 2002, before a twelve-year gap where no academic literature was published on the topic of additively manufactured immobilisers for radiotherapy. One journal article was then published in 2014, and literature has been consistently published in the range of 3–5 annual articles between 2015 and 2018. Overall, 55% (n = 10) of publications were journal articles, 28% (n = 5) posters, 11% (n = 2) conference papers and one thesis.

Summary information for each article is provided in Table 1, with an expanded table of results included in Supplementary Table S2. Sixty one percent (n = 11) of studies involved human tests, while 22% (n = 4) involved animals, 11% (n = 2) involved phantoms, and one was experimental without subject testing. The head and neck regions of the body were the most studied with 78% (n = 14) of articles, one of which focussed on the oral region, while 11% (n = 2) immobilised the whole body (of animals), one study focussed on the breast and one was experimental utilising test pieces only.

**Table 1:** Summarised results of the literature review categorised by body part and test subject.

Body part	Test Subject	Reference	Results
Head	Human, n = 1	Sanghera et al. [11]-2002	The 3D printed face mask exhibited similar performance under X-rays to traditional polystyrene based masks.
	Human, n = 1	Laycock et al. [10]-2015	Confirmation that 3D printed masks are feasible for treatment with similar qualities to Orfit masks.
	Human, n = 8	Unterhinninghofen et al. [29]-2015	The final product reportedly had high positioning accuracy equal to, or better than, traditional devices.
	Human, n = 10	Chen et al. [30]-2016	The proposed segmentation method can produce masks with high accuracy, with a small segmentation mean error of 0.4 mm.
	Human, n = 0	Márquez-Graña et al. [9]-2017	Authors claim that the proposed immobilizer is less invasive and more comfortable than a thermoformed or surgical immobiliser.
	Human, n = 17	Robertson et al. [31]-2017	The external reproducibility of 3D printed beam directional shells and thermoplastic equivalents had no considerable differences.
	Human, n = 11	Pham et al. [32]-2018	The 3D printed head was accurate enough to be used for moulding the thermoplastic masks onto.
	Human, n = 30	Luo et al. [33]-2018	3D printed headrests had higher transmittance (98.89%) compared with standard SRS headrests (98.51%).
	Human, n = 8	Haefner et al. [34]-2018	3D printed masks provided a high setup accuracy.
	Phantom, n = 1	Fisher et al. [35]-2014	A lack of adequate fitting of the 3D printed mask was found.
	Phantom, n = 1	Sato et al. [36]-2016	3D printed masks have almost the same positional accuracy to that of conventionally made devices.
	Animal, n = 10	Zarghami et al. [37]-2015	The mean irradiation targeting error was 0.14 ± 0.09 mm.
	Animal, n = 7	Slater et al. [38]-2016	Surgical head posts were found to reduce movement more than the masks.
Whole body	Animal, n = 6	McCarroll et al. [39]-2015	The 3D printed model decreased the setup variation considerably.
	Animal, n = 3	Steinmetz et al. [40]-2017	Animals were not distressed while they were immobilised.
Oral	Human, n = 1	Wilke et al. [41]-2017	3D printing eliminated several time-consuming steps in the fabrication of oral stents, minimising treatment delays.
Breast	Human, n = 10	Chen et al. [42]-2017	The 3D printed breast immobiliser considerably reduced the radiation exposure to the lungs and heart.
Experimental	Experimental	Meyer et al. [43]-2018	The effect on dosimetry requires investigation before clinical implementation of a 3D printed immobiliser. This study provides a framework for completing this testing.

Data captured from the studied articles showed that a range of technologies were used to capture geometry for designing the immobilisers, with 45% (n = 8) utilising CT scans, 22% (n = 4) MRI, 11% (n = 2) optical 3D scanning, and 22% (n = 4) undisclosed or not utilising any form of scanning. For the head and neck specifically, CT scans were used in 36% (n = 5) of studies, MRI was used in 29% (n = 4) of studies, optical 3D scanning was used in one study, and 29% (n = 4) were undisclosed or did not use scans to capture geometry.

Data captured from the literature also reveals that 78% (n = 14) of studies used 3D printing to fabricate the end-use immobiliser or headrest, while 11% (n = 2) used 3D printing to produce a replica of a head over which a traditional thermoformed mask immobiliser was created [32], [38], and one study used 3D printing to produce several immobilisers as well as a head for thermoforming a traditional mask over [10]. Six of the studies examined the accuracy of the 3D printed immobilisers, with four studies finding a good level of accuracy in face masks on human volunteers [29], [30], [31], [34], and one study finding a high degree of accuracy compared to the initial CAD model [11]. However, one study performed on a phantom found discrepancies in face mask geometry between 4 and 14 mm [35] and would not be suitable for immobilising a patient. This failure was attributed to improper thresholding values when converting the CT scan of the phantom into a 3D model.

The results in Table 1 (and S2) also reveal that studies analysing the setup accuracy of 3D printed immobilisers found a high degree of accuracy locating patients for treatment and reducing movement. In particular, several studies found that 3D printed immobilisers provided better accuracy than traditional methods [29], [33], [39], while others found a good level of accuracy that may be comparable to existing thermoformed methods [34], [36], [37], [42]. Only one study comparing surgical head posts to vacuum formed masks produced over 3D printed heads (moulds) for monkeys found the surgical head posts provided better accuracy by reducing movement during treatments [38]. However, this study also found that the addition of a mouth opening in the vacuum formed mask to allow food rewards encouraged voluntary engagement by the monkeys, reducing stress and anxiety. Studies also suggested that 3D printed immobilisers improve targeted treatment of tumours and reduced the radiation exposure to surrounding tissue [40], [42], although further research is required to confirm this hypothesis.

In relation to the beam attenuation of 3D printed materials, several studies found similarity with traditional thermoplastic immobilisers when made of a similar thickness [10], [11], [39]. Within a study on headrests for Stereotactic Radio Surgery (SRS), 3D printed headrests had higher transmittance (98.89%) than standard SRS headrests (98.51%) [33]. Two studies demonstrated a methodology to measure beam attenuation with variable thickness and infill patterns [10], [43].

Summarising the literature on 3D printed immobilisation devices, the identified advantages of the technology included: improved patient comfort [9], [29], [34], [37], reduced patient visits to a clinic [32], [35], [41], elimination of the stressful thermoforming process for face masks [29], [31], [34], [40], high accuracy and tolerance to the patient [11], [29], [30], [31], [34], repeatable positional accuracy [29], [33], [34], [36], [39], [42], less damage to surrounding healthy tissue [40], [42], similar beam attenuation properties to traditional polymer masks [10], [11], [33], [39] and the opportunity to consider additional features to improve fit or patient engagement [37], [38]. Disadvantages that were identified in the literature included: the potential for the 3D printing process to negatively influence the material behaviour of a device [30], inaccuracies due to conversion of scan data (e.g. CT) to 3D model [35], the slow process of 3D printing which is unsuitable for rapid deployment or modification [11], [32], lower accuracy compared to surgical immobilisation [38] and costs that may be the same or higher than traditional immobilisers [38].

**Fig. 1:**
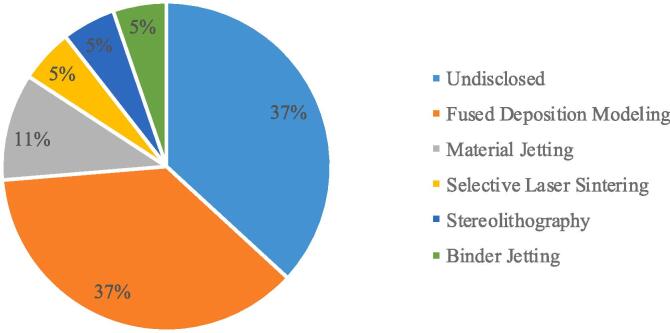
AM technologies utilised in literature.

From a technical perspective, Supplementary Table S3 summarises the details of additive manufacturing technologies, materials and software in the literature, which is important for future research planning in this field. According to ISO/ASTM 52,900 standards for classifying AM technologies, there are seven broad categories, five of which are represented in the eighteen papers collected in this study. As shown in Fig. 1, Fused Filament Fabrication (FFF) is the most utilised of the AM technologies. It is important to note that 37% (n = 7) of studies did not specify the print technology, making future replication of studies difficult.

## 4 Discussion

During radiotherapy, high doses of radiation are delivered to specific localised areas of the patient; as a result, targeting accuracy is vital in order to minimise negative effects to surrounding healthy tissue [7], [34]. Immobilisation devices are critical to this treatment process, minimising patient movement, and ensuring repeatability of the treatment over as much as 40 sessions [30].

While there is a general consensus that 3D printed immobilisers are a promising replacement for traditional immobilisers, particularly thermoformed face masks, the low volume of studies, and the low number of human, animal or phantom subjects included in the studies, means that further research is required to validate claims of providing improved performance or comfort. In total, 97 human patients and volunteers have featured in eleven studies, which is on average around nine subjects per study. This remains a small sample size and further research on human subjects is required in order to properly evaluate 3D printed immobilisers.

Given that research has only gained momentum in the last five years, it appears that academics and medical practitioners are still in the early experimental phase of developing 3D printed immobilisation technology, and this recent growth may be due to several factors: Firstly, costs of 3D printers and their materials have rapidly declined as the technology has become more mainstream [46], [47], making them accessible to researchers within medical and other disciplines [48]. Secondly, 3D printing technology has shifted from a predominantly rapid prototyping technology to an end-use manufacturing technology as materials have matured, making the production of functional parts, such as immobilisation devices, possible. A similar growth in medical research using AM technology was noted in a systematic review of surgical orthopaedic guides [48], with an increase in research observed shortly after 2009 when key FFF patents expired. FFF is an extrusion process also known as Fused Deposition Modelling (FDM), and has become synonymous with affordable desktop machines, although there are also high-end commercial varieties of this technology [44], [45]. A broader systematic review of 3D printing across medical fields confirmed this trend [49], and while little evidence exists to correlate the patent expiry with the increased use of FFF within research, the results from this study align with recent systematic reviews that indicate a rapid growth in medical research utilising 3D printing within the last ten years [48], [49].

However, the twelve-year period without any new research into 3D printed immobilisation devices between 2002 and 2014 is interesting, particularly because the results of the 2002 study were positive [11]. Several factors may account for this gap, including the relatively niche application of 3D printing for immobilisation compared to other aspects of medical 3D printing, such as surgical guides and implants, which dominate practice and literature. The technologies required for an immobiliser were also expensive and slow, for example the 3D scanning system in the 2002 study reportedly cost ~£100,000 [11], and the 3D printing machine was similarly expensive with a maximum print speed of 20 mm/s requiring five days to produce a single mask. It is only recently that costs have rapidly declined in line with Moore’s Law [47], [50], with today’s cheap ~$1000 desktop FFF machines capable of speeds of ~ 100 mm/s for the same material and layer thickness. Lastly, the large size of immobilisation devices makes them more challenging to 3D print than smaller medical devices like implants and surgical guides, which can be produced in a matter of hours and on a broader range of machines.

These factors remain challenging, with the first stage of creating a patient-specific immobilisation device requiring patient geometry to be digitised. Studies have used CT, MRI, optical 3D scanning and more manual CAD methods, with no consensus about the optimal method at this time. However, utilisation of CT scanners for capturing the digital data of the patient’s anatomy may not be the best technique as it increases the absorbed radiation dose by the patient if several CT scans are required. Designers also need to deal with the poor resolution of the CT scanners [51] compared to using high-quality optical scanners which are safer for patients and provide higher resolution of the external patient anatomy. While CT scanning was used in nearly half of the studies, given the relatively small number of studies in this review, and the focus on the end product rather than the technical aspects of digitising patient anatomy, further research is required to specifically examine and quantify the optimal method for digitising patient data for the development of an immobilisation device. It will become increasingly important for practitioners to learn how to manipulate DICOM files within 3D CAD software to progress this field of research, and may require collaborations between clinicians and surgeons with expertise in human anatomy, with designers and engineers with expertise in advanced CAD software systems.

The second phase of the workflow requires a digital design of an immobilisation device to be developed for each specific patient. From the results in Supplementary Table S3, it is clear that there is even less consensus about this stage, with a variety of software being implemented that ranges from high-end commercial design programs used by engineers and designers, to freely available software. Supplementary Table S3 also highlights that many studies do not disclose the software used to develop immobilisation devices, which may limit progress in this field as researchers and engineers are left to trial various methods rather than building upon those established across the literature. It is the recommendation of this research that future studies more rigorously report the details of the design process in order to establish protocols that others may build from, with scope for studies specifically examining the suitability of different CAD systems for the purposes of integrating patient scans with the design of custom immobilisers.

The final stage of the workflow is the production of an immobilisation device or mould using 3D printing. However, the printing quality of the final product needs to be evaluated through inspection for defects and radiologic suitability. One of the challenges in 3D printing is the inherent uncertainty which exists in the process of 3D printing. The final printed products could have different densities even if they are printed with the same printer and the same material from the same manufacturer, which could eventually lead to a variation in radiologic properties and dose uncertainty of the printed components [52].

Fig. 1 identified that FFF is the most utilised 3D printing method in the literature, which is most likely due to the affordability of machines. However, there is a breadth of FFF technologies used, from industrial machines [29], [34] which costs tens of thousands of dollars, to desktop machines costing a few thousand dollars [39], [40]. These differences, combined with the different materials being used, and the four other 3D printing technologies found in the literature, makes it challenging to objectively compare results since each technology, and each individual 3D printer, plays a significant role in the quality of the 3D printed outcome. Future research must aim to assess the most appropriate 3D printing technologies and materials for patient immobilisation, and converge towards standardised methods of producing immobilisers that are quantifiable and reproducible. The large number of studies that do not disclose the 3D print technology or material at this time inhibits this agenda, and researchers should prioritise this data alongside data related to the results of patient trials.

The results of this study also highlight gaps in knowledge surrounding the costs of 3D printed immobilisation devices compared with traditional methods. One study claimed a cost of ~$350CAD to produce a whole body immobiliser for a mouse [37], whereas another study with monkeys reported an initial setup cost of ~$3223USD, including the 3D printing of a head (mould) and vacuum forming of an immobiliser [38]. Each additional mask would then only cost ~$212USD since the mould could be re-used, although the study also found that over the course of a year, three of the monkeys required 1–3 replacement moulds and immobilisers due to changes in their body weight and head geometry. These costs are comparable to surgical methods [38]. Costs were not recorded in other literature, and it is unclear how economical 3D printed immobilisers are compared to other methods, especially across the range of print technologies and materials employed in literature to-date.

Production time also remains unclear, with the 2002 study claiming five days to 3D print a mask [11], while a more recent 2018 study recorded a 36 h print time for a human head in order to thermoform a mask over [32]. These times are significantly longer than more immediate traditional methods of thermoforming or surgically attaching immobilisers, however, a lack of information reported in literature does not provide enough evidence to provide a clear understanding of the time to 3D print immobilisation devices.

In conclusion, this literature review found that additive manufacturing technology has gained attention for the production of immobilisers for use in radiotherapy, emerging over the last five years as 3D printing technology has become more affordable, accessible and capable of producing functional parts. Research has found that the main advantages of the technology include the ability to manufacture immobilisers from digital patient data, removing the uncomfortable process of thermoforming directly over a patient, or surgically attaching fixation devices. Good levels of accuracy have also been reported, both in terms of matching the patient’s unique body geometry, as well as allowing for repeatable setup for treatment. Through the systematic review process, this study also highlighted several areas for future research, in particular the need for larger samples sizes in human testing in order to validate claims supporting the use of 3D printing to produce immobilisation devices. Furthermore, a lack of published information was noted in relation to the technical aspects of additively manufacturing immobilisers including their design, cost, production and associated settings, as well as materiality. These issues must be addressed as researchers continue to pursue this application of additive manufacturing.

## Declaration of Competing Interest

The authors declare that they have no known competing financial interests or personal relationships that could have appeared to influence the work reported in this paper.
