# Supplementary material for: A review of 3D printed patient specific immobilisation devices in radiotherapy
Source: Phys Imaging Radiat Oncol. 2020 Mar 20;13:30–5. doi: 10.1016/j.phro.2020.03.003 (PMC7807671; doi:10.1016/j.phro.2020.03.003)
Supplement: Supplementary data 1 [file mmc1.docx]

**Supplementary materials**

**Supplementary Fig. S1.** Key stages of the search strategy

**Supplementary Fig. S2.** Types of publications graphed by year of their publication

**Supplementary Table S1.** The investigated databases and the number of articles identified during the search phase

| **Database/Journal** | **Search Results** | **Database/Journal** | **Search Results** |
| --- | --- | --- | --- |
| Google Scholar | 800 | 3D Printing in Medicine | 13 |
| Pubmed NCBI | 680 | SpringerOpen | 54 |
| Scopus | 13 | Brachytherapy | 39 |
| Sage Journals | 300 | Korean Journal of Radiology | 2 |
| Radiation Oncology Journal | 39 | British Journal of Radiology | 172 |
| Radiotherapy and Oncology Journal | 12 | Japanese Journal of Radiology | 10 |
| Science Direct | 84 | Indian Journal of Radiology and Imaging | 222 |
| International Journal of Radiation Oncology | 864 | Polish Journal of Radiology | 0 |
| Practical Radiation Oncology | 127 | Journal of Radiology Case Reports | 2 |
| European Journal of Medical Physics | 137 | Iranian Journal of Radiology | 0 |
| Journal of Radiotherapy | 18 | The Egyptian Journal of Radiology and Nuclear Medicine | 28 |
| Journal of Oncology | 11 | Journal of Radiology Nursing | 9 |
| Journal of Clinical Oncology (ASCO) | 29 | Hong Kong Journal of Radiology | 1 |
| Journal of Radiotherapy in Practice | 44 | South African Journal of Radiology | 2 |
| IEEE Explore | 112 | Chinese Journal of Radiology | 0 |
| European Journal of Radiology | 9 | Iranian Journal of Medical Physics | 1 |
| Oncotarget | 4 | Polish Journal of Medical Physics and Engineering | 5 |
| Journal of Applied Clinical Medical Physics | 62 | Japanese Journal of Medical Physics (Igakubutsuri) | 5 |
| Journal of Medical Physics | 220 | Medical Dosimetry | 22 |

**Supplementary Table S2.** Scope, testing and results of literature (*full thesis unavailable)

| **Body part** | **Test Subject** | **Reference** | **Printed device** | **Treatment scenario** | **Design based on** | **Study objectives** | **Test method** | **Additional features** | **Results** |
| --- | --- | --- | --- | --- | --- | --- | --- | --- | --- |
| Head | Human, n=1 | B. Sanghera et al. [11]-2002  Journal | Facemask | --- | S4M Instantaneous 3D Surface Scanner | - Develop a 3D printed head immobiliser based on 3D scan data. - Compare the proposed technique with Plaster of Paris technique. - Investigate the beam attenuation effect. | A 3D printed mask is compared to a traditional polystyrene thermoformed mask under X-Ray dosing as well as critical dimensional accuracy. | --- | - The 3D printed face mask exhibited similar performance under X-rays to traditional polystyrene based masks. - The mask was made in 5 days. - Face mask was 3mm thick. - The 3D printed mask had a tolerance of ±0.3mm to the CAD model. |
|  | Human, n=1 | S. Laycock et al. [10]-2015  Journal | Facemask | --- | MRI using 1.5 GE Magnet combined with a whole-body CT scan. | - Construction of 3D printed masks across several 3D printers and materials for comparison. - Construction of a standard thermoformed mask from a 3D printed head for comparison. - Dosimetric characterisation of the 3D printing materials. | Dosimetric characterisation was performed by putting 3 different materials in different depths of water and investigating the change in radiation dose. | --- | - 2 mm 3D printed materials produce lower attenuation than the 2.4mm Orfit mask. - All the materials produce a small decrease (up to 1%) in the dose to the tumour due to attenuation of the beam. - The skin dose for the 2mm 3D printed materials is similar or lower than the 2.4mm Orfit mask. - Confirmation that 3D printed masks are feasible for treatment with similar qualities to Orfit masks. |
|  | Human, n=8 | R. Unterhinninghofen et al. [29]-2015  Poster | Facemask | --- | MRI using Siemens Avanto 1.5 T | - Develop a novel approach to create customised fixation medium. - Investigate the positional accuracy on 8 volunteers. | Positional accuracy of devices on volunteers assessed through an automatic image-to-image registration. | An interface was added to the mask for fixing it to an intervention table | - The mask was produced in a fully contact free manner using the MRI scans, improving patient comfort. - The final product reportedly had high positioning accuracy equal to, or better than, traditional devices. - Mean overall displacement vector lengths of 1.20mm between mask and patient. |
|  | Human, n=10 | S. Chen et al. [30]-2016  Conference | Head and neck mask | --- | Cone-beam CT scan | - Develop a fully automatic framework for making 3D printed head and neck immobilisation masks by implementing 3D printing and image analysis techniques. | The model’s feasibility was evaluated through assessing the accuracy of segmentation and facial feature recognition accuracy | --- | - The proposed segmentation method can produce masks with high accuracy, with a small segmentation mean error of 0.4mm. - Feature recognition had a mean registration error of 11.78mm. - Evidence of an automatic system to create facial masks using 3D printing. |
|  | Human, n=0 | C. Márquez-Graña et al. [9]-2017  Conference | --- | Stereotactic radio surgery (SRS) | --- | - A feasibility study was performed for a new head support and device design following ISO 13485 protocols. | --- | --- | - Authors claim that the proposed immobilizer is less invasive and more comfortable than a thermoformed or surgical immobiliser, however, the sketch designs are concepts and have not been manufactured or trialled. |
|  | Human, n=17 | F. M. Robertson et al. [31]-2017  Thesis* | Beam directional shell | External beam radiation therapy | --- | - Evaluate the feasibility of 3D printed Beam Directional Shells (BDSs) by comparing them with traditionally made thermoplastic BDSs. - Evaluate patient anxiety during testing. | 3D printed and traditionally made BDSs was tested on 17 healthy volunteers. Over four sessions, the left-right, superior-inferior, anterior-posterior rotations and alignments of the shells were assessed through the retrospective isocentre positioned on the shells. The obtained data was also evaluated for its homogeneity and normality. | --- | - The external reproducibility of the 3D printed BDSs and thermoplastic BDSs had no considerable differences. - The thermoplastic BDSs were a more stressful experience for the patients than 3D printed BDSs. |
|  | Human, n=11 | Q. V. V. Pham et al. [32]-2018  Journal | Patient’s head model | Whole brain radiotherapy (WBRT) | Cone-beam CT scan | - Assess the accuracy of a 3D printed model of a patient’s head and neck for thermoforming a mask over. | The accuracy of the printed head in terms of volume was compared to the original head, (co-registration). Dosimetric alterations due to the translational and rotational differences were also evaluated. | --- | - The 3D printed head was accurate enough to be used for moulding the thermoplastic masks onto. - 3D printing the head would take 36 hours which would not be practical for patients that need radiation therapy in one to two days. |
|  | Human, n=30 | L. Luo et al. [33]-2018  Poster | Head rest | Stereotactic radio surgery (SRS) | --- | - Develop a 3D printed SRS headrest for paediatrics. - Compare the transmittance and setup errors of standard SRS headrests and 3D printed ones. | Two groups of paediatric patients were tested. One group used 3D printed headrests with masks, the other group was immobilised using standard SRS headrests with masks. | --- | - 3D printed headrests had higher transmittance (98.89%) compared with standard SRS headrests (98.51%). - For the group with 3D printed headrests, the setup errors in AP, VRT and LAT directions were, (0.10 ± 0.07) mm, (0.04 ± 0.055) mm, and (0.04 ± 0.055) mm respectively. For the group with standard SRS headrests, the setup errors were (0.1 ± 0.1) mm, (0.16 ± 0.055) mm, and (0.08 ± 0.11) mm respectively. Therefore, the 3D printed headrests had less setup errors. |
|  | Human, n=8 | M. F. Haefner et al. [34]-2018  Journal | Facemask | --- | MRI | - Develop an approach for generating 3D printed patient specific immobilisers based on MRI data. - Investigate device accuracy in positioning the patients for treatment. | 8 volunteers were immobilised using 3D printed masks and headrests and their head displacements were measured with MRI for 10 simulated radiotherapy fractions. These were compared to their first MRI images using an automatic image-to-image registration. | An interface for the coach was added to the mask designs. | - 3D printed masks provided a high setup accuracy: - The absolute lateral (x), vertical (y) and longitudinal (z) translations ranged between −0.7 and 0.5 mm, −1.8 and 1.4 mm, and −1.6 and 2.4 mm, respectively. - The absolute rotations for pitch (x), yaw (y) and roll (z) ranged between −0.9 and 0.8°, −0.5 and 1.1°, and −0.6 and 0.8°, respectively. - The mean 3D displacement was 0.9 mm with a standard deviation of the systematic and random error of 0.2 mm and 0.5 mm, respectively. |
|  | Animal, n=10 | N. Zarghami et al. [37]-2015  Journal | Mouse head holder | Focal radiation therapy | --- | - Develop a customised mouse head restraint for accurate focal radiotherapy. - Evaluate the reproducibility of the imaging. - Evaluate the accuracy of the device. | During the test procedure the mean irradiation targeting error was computed and rotation between the mouse head and the beam axis was also measured. | Ear bars, tooth block | - Mouse was not distressed during the fixation procedure using ear bars and tooth block. - The 3D printed immobiliser cost ~$350CAD using 263g VeroWhite Plus and 129g supporting material. - The mean irradiation targeting error was 0.14 ± 0.09mm - The rotation between mouse head and beam axis was reported 1.2^o^ ± 1.0^o^. - The radiation beam edge could be located within 0.15mm of the intended target. - To overcome the fragility of the 3D printed material, the base of the head holder was designed thicker (1.5 cm) compared to the other parts. |
|  | Animal, n=7 | H. Slater et al. [38]-2016  Journal | Animal’s head model | Neuroscientific procedures | MRI | - Develop a new head immobilisation system for monkeys with an automated voluntary engagement option. | Each monkey’s head was 3D printed from the MRI and a thermoplastic mask was vacuum formed over it with 4mm PETG, including an opening for food rewards. This was compared to a mask produced using plaster bandages and alginate, with a similar thermoformed mask produced over it. | A mouth access is considered for the device for receiving rewards | - The initial setup for the helmet production, including equipment and consumables, was $3223 USD. After the initial equipment investment, a large number of helmets can be produced at a cost of approximately $212 per helmet. In comparison, a single implant procedure costs ∼$2930, with additional costs of ∼$202 for each implant maintenance procedure. - Over the course of a year, three animals on study with the helmet system required 1–3 replacements due to their change in body weight. - Surgical head posts were found to reduce movement more than the masks (mean movement in helmet: 1.05mm; mean movement with head post: 0.53mm). - Face masks with food reward allowed for monkey’s to be trained for voluntary engagement. |
|  | Phantom, n=1 | M. Fisher et al. [35]-2014  Journal | Head and neck shell | External beam radiation treatment | GE Medical Systems Optima CT Scanner | - Preclinical assessment of a new fixation system by measuring the mask’s quality of fit. | The quality of fit is evaluated by computing the distance between the mask’s inner surface and the phantom’s outer surface using an error analysis tool developed by the authors. | --- | - A lack of adequate fitting of the mask was found with >80% of slices showing a gap between phantom and mask of ≤4 mm, and some areas as much as 14 mm. - The authors suggest improper thresholding during processing of the CT scan as the main error source. |
|  | Phantom, n=1 | K. Sato et al. [36]-2016  Poster | Immobilization mask and head rest | --- | Cone-beam CT scan | - Evaluate the feasibility of a 3D printed immobilisation device. - Investigate its positional accuracy in comparison with conventionally manufactured masks. | A 3D printed mask was compared to a thermoformed mask by putting these masks separately on the same phantom head and computing the positional deviations between coordinate origins in X-ray images. | --- | - Additively manufactured masks have almost the same positional accuracy to that of conventionally made devices. - The lateral, vertical and lengthwise displacement for an additively manufactured mask was (mean [standard deviation]): 0.31 [0.27], -0.28 [0.09], and -0.02 [0.08] respectively. For traditionally made devices results were: 0.84 [0.27], 0.29 [0.06], and 0.03 [0.14] respectively. - For an additively manufactured device, in role, pitch and yaw directions, the rotational shift was (mean [standard deviation]): 0.08 [0.74], -0.31 [0.08], and 0.62 [0.13] respectively. For traditionally made devices results were: 0.17 [0.67], -0.09 [0.06], and 0.15 [0.17] respectively. |
| Whole body | Animal, n=6 | R.E. McCarroll et al. [39]-2015  Journal | Whole body immobilization device | --- | Cone-beam CT scan | - Develop a technique to create a 3D printed small animal immobiliser.   Reduce the inter-fraction rotational variation. | Translational displacement in the fixation device was evaluated and beam attenuation was assessed using a farmer-type ionization chamber. | --- | - The 3D printed model decreased the setup variation considerably. In rotational displacement, the average reduction was 87% ± 3% in roll, 76% ± 3% in pitch and 78% ± 3% in yaw. A translational displacement of less than 1.5 ± 0.3mm was also reported. - The attenuation percentage of each 3 test ABS-specimens was 98% ± 1%.   The ABS material was compatible with MRI imaging and no artefact was produced. |
|  | Animal, n=3 | A. Steinmetz et al. [40]-2017  Poster | Whole body immobilization device | Proton and photon radio therapy | Cone-beam CT scan | Develop a 3D printed immobilisation device for precise tumour targeting. | A 3D printed immobiliser was fitted to mice with a subcutaneous flank tumour (EL4 Thymoma). The tumour movement was observed over 15 minutes of video monitoring. | A brass block is considered for the device to decrease the beam penumbra | - Animals were not distressed while they were immobilised.   Proton and photon treatment plans were generated using Eclipse software, demonstrating uniform targeting of the tumour with <10% of the prescribed dose delivered to the abdominal or thoracic cavity. |
| Oral | Human, n=1 | C. T. Wilke et al. [41]-2017  Journal | Oral stent | --- | Cone-beam CT scan | - Develop a technique for generating oral stents through CT images and 3D printing.   Reduce the need for dental and oral specialists for making these useful devices. | For checking the proper positioning of the occlusal surfaces in the desired region the dentition was overlaid with a rectilinear volume template. | --- | - Initial image segmentation to the completion of the post-processing steps completed in less than eight hours. - Typically, oral stent fabrication requires at least 2 appointments with the patient to obtain dental impressions and assess the fit of the device. This method eliminates several of these steps and thus minimises treatment delays.   A mixture of proprietary and open-source/free software had to be used; there is no single software solution for this process. |
| Breast | Human, n=10 | T. Chen et al. [42]-2017  Poster | Breast holder | Whole breast irradiation (WBI) | OpenNI-compatible scanner | - Develop a patient specific breast holder using 3D printing.   Protect heart and left anterior descending artery from radiation exposures. | Dosimetric parameters of breast treatment without an immobiliser was compared to treatment with a 3D printed immobiliser in 10 patients. A follow up treatment with the 3D printed immobiliser was compared with earlier treatment to compare setup accuracy. Paired sample T-test was used to determine statistical significance. | --- | - Setup time for each patient was <5min. - The 3D printed breast immobiliser considerably reduced the radiation exposure to the lungs and heart: Set 1 (no immobiliser) vs Set 2 (3D printed immobiliser) mean heart dose: 478.4 ± 58.6 vs 346.4 ± 58.9 cGy; mean LAD dose: 1751.1 ± 217.8 versus 1427.3 ± 242.2 cGy; left lung V_10_ : 22.1 ± 0.9 vs 16.8 ± 1.1 %; left lung V_20_ : 16.7 ± 0.8 vs 11.3 ± 0.9 %, all p<0.01.   Setup errors between Sets 2 and follow-up Set 3 were 0.67 ± 0.16, 0.54 ± 0.15, and 0.23 ± 0.14 cm in vertical, lateral and cranial axes, respectively. |
| Experimental | Experimental | T. Meyer et al. [43]-2018  Journal | --- | --- | Cone-beam CT scan | Propose a workflow for dosimetric characterisation of 3D printing materials using samples, while the dimensionality of the final product (immobilisation device) is not known. | Wedge and cylinder geometries were used as test samples. A case study with a cranio-spinal irradiation immobiliser was also considered with known but adaptable dimensions. | --- | - The relationship between shell thickness and infill density as they contribute to physical density vary depending on the printed device. - The effect on dosimetry requires investigation before clinical implementation of a 3D printed immobiliser. This study provides a framework for completing this testing.   Beam attenuation increased as the sample wedge thickness increased. |

**Supplementary Table S3.** Technical fabrication details of immobilisers (*full thesis unavailable)

| **AM Technology** | **Material (Type)** | **Reference** | **Printer (Type)** | **Software** |
| --- | --- | --- | --- | --- |
| Fused Filament Fabrication | ABS 400 (filament) | B. Sanghera et al. [11]-2002, Journal | Stratasys RP 1650 (FFF) | CAD: Unspecified.  3D Printing: Stratasys proprietary software. |
|  | ABS (filament) | R.E. McCarroll et al. [39]-2015, Journal | MakerBot Replicator 2X (FFF) | --- |
|  | ABS (filament) | M. F. Haefner et al. [34]-2018, Journal | Dimension SST1200es (FFF) | CAD: In-house developed software for the mask.  CAD: Undisclosed commercial software for the headrest. |
|  | PLA (filament) | A. Steinmetz et al. [40]-2017, Poster | Ultimaker 2 (FFF) | CAD: Blender |
|  | PLA (filament) for head and neck rest | Q. V. V. Pham et al. [32]-2018, Journal | Big Builder Dual-Feed (FFF) | CT to Mesh: 3D Slicer  CAD: CATIA  Surface Segmentation: Eclipse |
|  | Onyx (chopped carbon fibre filament) | T. Meyer et al. [43]-2018, Journal | Markforged Onyx One (FFF) | --- |
|  | --- | R. Unterhinninghofen et al. [29]-2015, Poster | Dimension SST1200es (FFF) | In-house developed software |
| Selective Laser Sintering | PA2200 (polyamide powder) | M. Fisher et al. [35]-2014, Journal | Formiga P110 (SLS) | --- |
| Binder Jetting | EOS PA 3200 (polyamide powder),  VisiJet Clear (resin) | S. Laycock et al. [10]-2015, Journal | Z-Corp 650 (BJ), Z-Corp 450 (BJ), Eden 250 (MJ) | CAD: Tommomask  Image Segmentation: Osirix |
| Material Jetting | VeroWhite Plus (acrylic resin) | N. Zarghami et al. [37]-2015, Journal | Objet 30 Pro (MJ) | CAD: AutoCAD 2014 |
| Stereolithography | Formlabs standard clear resin | C. T. Wilke et al. [41]-2017, Journal | Formlabs Form 2 (SLA) | Image Segmentation: Velocity Oncology Imaging Informatics System  CT to Mesh: 3D Slicer  CAD: Autodesk Meshmixer  3D Printing: PreForm |
| Undisclosed | ABS (resin) | K. Sato et al. [36]-2016, Poster | --- | --- |
|  | Thermoplastic elastomer | T. Chen et al. [42]-2017, Poster | --- | Image Segmentation: In-house developed image processing software  Treatment Planning: Pinnacle |
|  | --- | S. Chen et al. [30]-2016, Conference | --- | --- |
|  | --- | H. Slater et al. [38]-2016, Journal | --- | MRI processing: Amide 3D |
|  | --- | C. Márquez-Graña et al. [9]-2017, Conference | --- | --- |
|  | --- | F. M. Robertson et al. [31]-2017, Thesis* | --- | --- |
|  | --- | L. Luo et al. [33]-2018, Poster | --- | --- |

Abbreviations: ABS = Acrylonitrile Butadiene Styrene, BJ = Binder Jetting, CAD = Computer-Aided Design, FFF = Fused Filament Fabrication, MJ = Material Jetting, PLA = Polylactic Acid, SLA = Stereolithography, SLS = Selective Laser Sintering
